# The influence of government policies on the nurse practitioner and physician assistant workforce in the Netherlands, 2000–2022: a multimethod approach study

**DOI:** 10.1186/s12913-023-09568-4

**Published:** 2023-06-06

**Authors:** Ellen J. C. M. Dankers-de Mari, Anneke J. A. H. van Vught, Hetty C. Visee, Miranda G. H. Laurant, Ronald Batenburg, Patrick P. T. Jeurissen

**Affiliations:** 1 Scientific Center for Quality of Healthcare (IQ Healthcare), Radboud University Medical Center, Radboud Institute for Health Sciences, P.O. Box 9101, 6500 HB Nijmegen, The Netherlands; 2 School of Health Studies, HAN University of Applied Sciences, P.O. Box 6960, 6503 GL Nijmegen, The Netherlands; 3 Regioplan, Jollemanhof 18, Amsterdam, 1019 GW The Netherlands; 4 Netherlands Institute for Health Services Research, NIVEL, P.O. Box 1568, 3500 BN Utrecht, The Netherlands

**Keywords:** Nurse practitioners, Physician assistants, Physician associates, Skill-mix, Policy, Workforce, Review, Survey

## Abstract

**Background:**

Many countries are looking for ways to increase nurse practitioner (NP) and physician assistant/associate (PA) deployment. Countries are seeking to tackle the pressing issues of increasing healthcare demand, healthcare costs, and medical doctor shortages. This article provides insights into the potential impact of various policy measures on NP/PA workforce development in the Netherlands.

**Methods:**

We applied a multimethod approach study using three methods: 1) a review of government policies, 2) surveys on NP/PA workforce characteristics, and 3) surveys on intake in NP/PA training programs.

**Results:**

Until 2012, the annual intake into NP and PA training programs was comparable to the number of subsidized training places. In 2012, a 131% increase in intake coincided with extending the legal scope of practice of NPs and PAs and substantially increasing subsidized NP/PA training places. However, in 2013, the intake of NP and PA trainees decreased by 23% and 24%, respectively. The intake decreased in hospitals, (nursing) home care, and mental healthcare, coinciding with fiscal austerity in these sectors. We found that other policies, such as legal acknowledgment, reimbursement, and funding platforms and research, do not consistently coincide with NP/PA training and employment trends. The ratios of NPs and PAs to medical doctors increased substantially in all healthcare sectors from 3.5 and 1.0 per 100 full-time equivalents in medical doctors in 2012 to 11.0 and 3.9 in 2022, respectively. For NPs, the ratios vary between 2.5 per 100 full-time equivalents in medical doctors in primary care and 41.9 in mental healthcare. PA-medical doctor ratios range from 1.6 per 100 full-time equivalents in medical doctors in primary care to 5.8 in hospital care.

**Conclusions:**

This study reveals that specific policies coincided with NP and PA workforce growth. Sudden and severe fiscal austerity coincided with declining NP/PA training intake. Furthermore, governmental training subsidies coincided and were likely associated with NP/PA workforce growth. Other policy measures did not consistently coincide with trends in intake in NP/PA training or employment. The role of extending the scope of practice remains to be determined. The skill mix is shifting toward an increasing share of medical care provided by NPs and PAs in all healthcare sectors.

**Supplementary Information:**

The online version contains supplementary material available at 10.1186/s12913-023-09568-4.

## Background

The first nurse practitioners (NPs) and physician associates/assistants (PAs) entered the Dutch labor market in 2000 and 2004, respectively [1]. The factors that contributed to the introduction of these professions included the increasing demand for care, regional medical doctor shortages, and the expected cost-effectiveness of NPs and PAs. This was combined with an accommodative policy that focused on the need for a university program for nurses [2, 3]. NP/PAs are perceived as professionals who can help alleviate healthcare challenges, because they can take over tasks from physicians.

The scopes of practice of NPs and PAs lie at the interface of the medical professions and result in task reallocation [4]. In healthcare practice, some tasks of NPs and PAs may overlap [5, 6]. However, they have different profiles and educational preparation (Table 1) [7]. In the Netherlands, universities of applied sciences are responsible for the content of educational programs, and such content is derived from the professional profiles. Training is at the master’s degree level. It includes a dual work-education model, with 1 day in school and 4 days of practical learning within the healthcare organization where the trainee works [1, 8].

**Table 1:** NP and PA profiles, tasks, and (pre)education in the Netherlands

	**NP profile**	**PA profile**
Positioning	‘Bridge officers’ between nursing and medical care	Medical professionals within a subfield of medicine
Setting	Can be deployed within one or a few patient groups (expert). Is best in (nurse) innovations and as a point of contact when many medical specialties are involved in treating an individual patient	Widely employable in medical settings where different disorders come together (multimorbidity)
Focus	Focus on improving nursing care and integrating this with medical care. The emphasis is on the biopsychosocial model	Focus on medical care, with an emphasis on the medical model and considering the biopsychosocial model
Tasks	General healthcare NPs spend most of their time on: 1) patient consultations and visits, 2) patient-related administration, 3) multidisciplinary consultation and coordination, 4) policy and project tasks, and 5) participation in working groups and committees	PAs spend most of their time on: 1) patient consultations and visits, 2) patient-related administration, 3) multidisciplinary consultation and coordination, 4) fulfilling the function of a ward doctor, and 5) guiding residents
Education	Training takes 2 years and is focused on general areas of competence (clinical expertise: nursing and medical, communication, cooperation, organization, health promotion, science, and professionality) and specialism-specific competencies related to the scope of practice	Training takes 2.5 years and is focused on general areas of competence (medical expertise, communication, cooperation, leadership, scientific thinking, health advocacy, professionalism) and competencies associated with medical specialization
Pre-education and work experience	Pre-education and work experience are relevant because it concerns a follow-up profession. To be admitted to NP training, a registered nurse must possess a relevant bachelor's degree (within healthcare) and have 2 years of relevant work experience	Pre-education and work experience can vary because it concerns retraining. To be admitted to PA training, an entrant must have completed higher vocational education in healthcare and have at least 2 years of practical experience in direct patient care at a higher professional education level

Looking back on the reasons for the introduction of both professions in the Netherlands, NPs and PAs do indeed fill part of the increasing demand for care [1]. Furthermore, several studies show that NP/PA deployment results in an equal or better quality of care, patient satisfaction, and comparable or reduced healthcare costs compared to medical doctor deployment [9–20]. In the USA, Veterans Affairs administrative data made clear that primary care patients reassigned to NPs experienced similar outcomes and incurred less utilization at comparable cost relative to MD patients [10]. For PAs, a systematic review showed that this efficiency was sometimes due to reduced labor costs and sometimes because they were more effective producers of care and activity [9]. Furthermore, some studies state that a (local) shortage in medical doctors incentivizes NP/PA employment [15, 21, 22].

Since the start of NP and PA training, various and sometimes unique policy measures have been taken in the Netherlands to facilitate task reallocation and further NP/PA training and deployment. To understand the policy measures, an explanation of the Dutch healthcare system is provided in Table 2. Policies include extending the legal scope of practice, creating reimbursement opportunities, and providing a national subsidy scheme for NP/PA training. The subsidy includes a partial reimbursement for salary costs for the replacement of NP/PA students and is received by the students’ employer [1]. Based on estimates of the future required NP/PA (training) capacity, the Ministry of Health, Welfare, and Sport decides the number of subsidized training places. Education for healthcare professionals is one of the public values the state regulates and preserves.

**Table 2:** The Dutch healthcare system

*Three principles underpin the Dutch healthcare system: access to care for all, solidarity through medical insurance (compulsory and available for all), and high-quality healthcare services. Private, not-for-profit health insurance cooperatives play a key role (1). Active purchasing by insurers incentivizes autonomous providers to perform better in terms of costs and quality, and consumers’ freedom of choice encourages insurers to offer competitive health plans. * *The state regulates healthcare to preserve public values (2).*	

Evaluation data on the impact of these policies on the training and employment of NPs and PAs are currently limited to evaluations of the subsidy scheme for the training programs for NPs and PAs [23–26]. In these evaluations, the universities of applied sciences state that the number of NPs and PAs would never have increased so much without subsidy schemes [24–26]. However, it is still unknown to what extent these policy measures affect the NP/PA workforce.

### Study aim

This study aims to analyze the effects of policy measures on NP/PA workforce development in the Netherlands. The research questions that will be addressed are therefore as follows:

1. Which policy measures were taken by the government to facilitate NP/PA training and deployment?
2. How did NP/PA training and employment and the NP/PA-medical doctor capacity ratio develop over time and in each setting?
3. To what extent did policy measures affect NP/PA training and employment?

## Methods

To answer the research questions, a multimethod approach consisting of a review and surveys was applied. To gain insight into which policy measures may have affected NP/PA training and employment, and the NP/PA-medical doctor capacity ratio, we analyzed whether the moment of implementation of policy measures corresponds with trends in the workforce size of the professions and the intake in NP/PA training courses.

### Review

We reviewed publications and reports to identify the relevant policy measures in the Netherlands between January 2000 and January 2022. Our focus was on changes in laws, regulations, and healthcare funding in healthcare sectors where NPs and PAs work and may affect these professions’ employment and training. The sources were government publications, publications of sectoral and professional organizations and research reports. We used two approaches to discover relevant information. First, we monitored documents and news items that have been published by professional associations of PAs, NPs, and medical doctors, the Consultation Platform NP/PA, the National Center of Knowledge for Task Reallocation in Primary Care, the National Healthcare Authority, the General Consultation Labor Market Policy in Healthcare and the Standing Parliamentary Committee on Health, Welfare, and Sport in the period from January 2014 to January 2022. Where relevant, references to earlier sources up to 2000 were included. We included sources regarding NPs or PAs containing the following terms: legislation, regulations, policy, subsidy schemes, financing of care, and reimbursement of care. Second, the overview was supplemented, and to complete it, policy measures and underlying publications were added by an NP/PA policy expert, professional associations, and the PA/NP committee within the Advisory Committee on Medical Manpower Planning (ACMMP), consisting of program coordinators of NP/PA training programs, NPs, PAs, and representatives of health insurers.

### Surveys of training programs

At the end of 2018 and the end of 2021, the chairpersons of the national training committees were asked to send all training programs a survey regarding the annual intake and number of graduates from the 2-year MANP (Master Advanced Nursing Practice) and 2.5-year MPA (Master Physician Assistant) training programs at the universities for applied sciences. The training programs started in 1997 and 2001, respectively. Data were collected from the 2000–2001 cohort until the 2021–2022 cohort. We also collected data at the sector level from 2012 onward. No personal data were collected.

### Surveys of alumni

Through surveys among alumni of all MANP and MPA courses at universities for applied sciences, we collected data on the labor market characteristics and executed tasks of NPs and PAs [5, 6, 27–33]. Training program graduates were invited to take part in the digital survey by e-mail in 2012, 2016, 2018, and 2021. Informed consent was obtained. No personal data were collected. The encrypted files, the decryption code and back-up data are stored securely.

We calculated the response based on the number of graduates at the time. Among NPs, there were 4,304 graduates from the MANP training programs at the end of 2021. Among PAs, there were 1,741 graduates. Alumni no longer working in the profession were also invited to complete the survey. We could not contact all alumni due to missing contact details. In 2022, there was contact information for 88% of MANP alumni and 96% of MPA alumni.

A weighting factor was constructed to extrapolate to the national level, based on the total number of persons per intake and the diploma year compared to the number of respondents from that year. In the 2018 (NP and PA) and 2021 (NP) data collection, we used an additional weighting factor to correct for alumni who were not working in the professions, as they were less likely to participate in the survey. This correction was based on the number of people registered in the Nurse Practitioner Register (NPs) and the Individual Health Care Professions register (NPs and PAs) and the share of inactive NPs and PAs in the registration of Statistics Netherlands. In 2021, no additional weighting factor was necessary for the PAs because: 1) the weighted number of respondents who worked as a PA did not exceed the number of registered PAs, and 2) the share of active PAs corresponded to the registration of Statistics Netherlands.

To gain insight into the influence of shortages in medical doctors who specialized after initial medical training on NP/PA training and employment, we also collected data on the capacity of medical doctors by medical field in terms of full-time equivalent (FTE) from 2013 onward. Medical doctors at a master’s degree level were excluded because their capacity was not a reason for implementing the NP/PA professions. To obtain these data we consulted reports from the ACMMP regarding the required training capacity of medical doctors [34].

## Results

### Policy measures

In the overview of recent decades of Dutch healthcare policy, the government and health insurers have implemented various policy measures that may have affected NP/PA training and employment. The policy measures relate to subsidies for NP/PA training, the funding of platforms and research, extending the scope of practice, legal acknowledgment, reimbursement regulations, and healthcare funding (see timeline Table 3) and followed after recommendations of the Committee Implementation Training Continuum and Task Reallocation in 2003 [35].

**Table 3:**
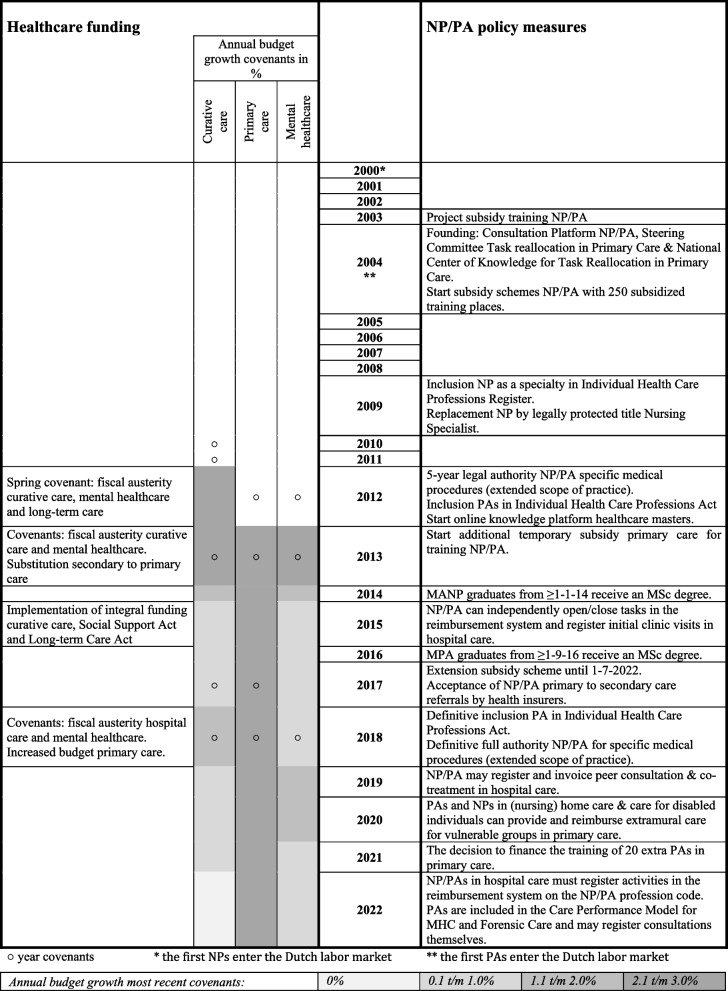
Policy measures regarding NPs and PAs and healthcare funding 2000–2022, January 1

### Training subsidies

Since 2004, the number of subsidized training places for NP and PA training has greatly increased. After 2013 the number of structurally subsidized places was fixed at 250 places for the MPA and 450 for the MANP. From 2013, general practices and out-of-hours primary care services could apply for additional funding [36]. In 2021, it was decided to finance training for 20 additional PAs within primary care [37].

### Funding platforms and research

Since 2004, the Dutch government has financed a knowledge center and consultation platforms on task reallocation. In addition, research on task reallocation was funded.

### Extending the scope of practice

NPs and PAs have been granted legal authority for specific reserved medical procedures. Here, extending the scope of practice concerns routinely performed medical procedures of limited complexity, in which the risks are reasonable to oversee and for which national guidelines, standards, and protocols apply. From January 1, 2012, the scope of practice was extended for five years [38, 39]. From September 1, 2018, the extended autonomous scope of practice of NPs and PAs for specific reserved medical procedures became permanent. NPs and PAs can independently indicate, execute and delegate several reserved medical procedures depending on their experience and specialism and following laws and regulations, i.e., surgical procedures, endoscopies, catheterizations, injections, punctures, elective cardioversion, defibrillation, and prescribing drugs that are available only by prescription [40–43].

### Legal acknowledgment

With the inclusion of NPs and PAs in the Individual Health Care Professions Register and Act in 2009 and September 2018, respectively, these professions obtained, among others, a protected professional title and governance by disciplinary law. Since January 1, 2014, and September 1, 2016, NP and PA graduates have received an MSc degree.

### Reimbursement regulations

Since 2015, NPs and PAs have been allowed to open and close tasks in the reimbursement system in hospital care [44]. As a result, they can carry out initial clinical visits independently in addition to follow-up visits. As of 2019, NPs and PAs may also register and invoice peer consultation and cotreatment activities in the reimbursement system under their name [45–47]. Starting in 2022, hospital care activities must be registered in the reimbursement system under the profession code of the healthcare professional providing care to promote task reallocation [48]. In (nursing) home care and care for disabled individuals, starting in 2019, it became possible for NPs and PAs to provide and reimburse care for vulnerable groups in primary care, similar to elderly or disabled care physicians [49–51].

### Healthcare funding

The Dutch government defines budgets for healthcare sectors where NPs and PAs operate. In the spring of 2012, agreements between the governing coalition and part of the opposition were made regarding an extensive program of fiscal austerity that would also affect curative care (among others, hospital care), long-term care (among others (nursing) home care), and mental healthcare [52]. In the following years, the government made covenants with sectoral and professional organizations regarding, among others, healthcare budgets (Table 3) [53–59].

Further background information about the policy measures can be found in Additional file 1: Appendix 1.

### The development of the Dutch NP/PA workforce

#### Intake in training programs 2001–2022

**Fig. 1:**
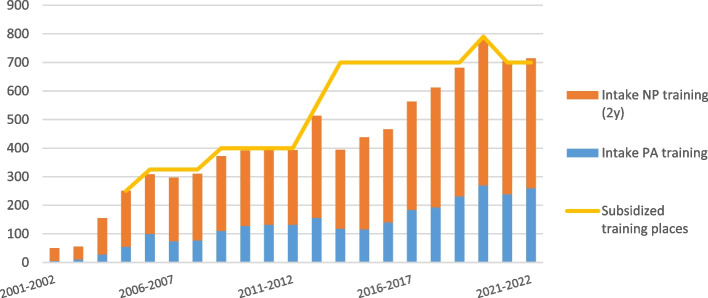
Annual intake in the PA and 2-year NP training programs 2001–2022

All NP and PA courses at the universities for applied sciences in the Netherlands (9 and 5, respectively) provided the information requested in the surveys. Figure 1 shows that the annual intake almost corresponded to the number of subsidized training places between the 2001–2002 and 2012–2013 cohorts, with a 131% increase in the 2012–2013 cohort [1]. In 2013–2014, the annual intake in NP and PA training programs decreased sharply by 23% and 24%, respectively. Starting with the 2014–2015 cohort, the yearly intake increased again and almost matched once again the number of subsidized training places in 2018–2019.

**Fig. 2:**
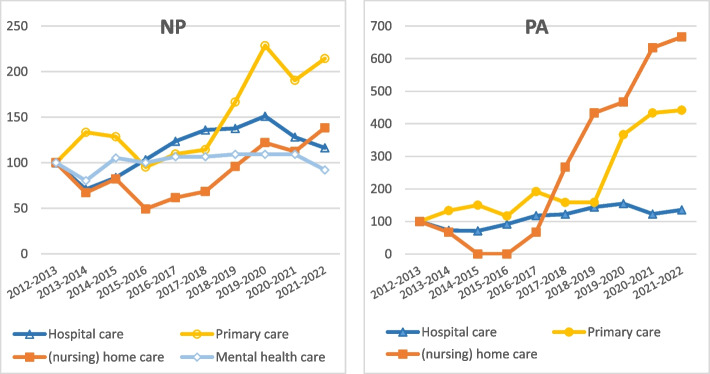
Annual intake in the training programs per sector, 2012–2022 (indexed, with 2012–2013 as the base cohort). Programs and sectors with an average of fewer than 15 entrants per year since 2017 have been excluded

The numbers of the annual intake by sector have been available since the 2012–2013 cohort [1]. In hospital care, PA and NP training intake decreased by 27% and 29%, respectively, between 2012–2013 and 2013–2014 (Fig. 2). In (nursing) home care and mental healthcare, the number of new students entering the NP program fell in 2013–2014 by 33% and 20%, respectively. The intake decreased in a large majority of the training programs. For hospital care, 11 out of 14 NP/PA programs (79%) were involved. For NP training in (nursing) home care and mental healthcare, the intake dropped by 67% and 73% of the programs with newcomers in the 2012–2013 and 2013–2014 cohorts, respectively. In contrast, the number of primary care newcomers increased in both PA and NP training programs by 33%. Nevertheless, the numbers in this sector were relatively small, with 16 (PA) and 28 (NP) newcomers in 2013–2014. The influx into NP and PA training in (nursing) home care shows a rising trend from 2015–2016. Since the 2018–2019 cohort, the inflow into PA training in primary care has also increased sharply.

#### NP, PA, and medical doctor capacity

In 2012, 2016, and at the end of 2018 and 2021, 45%, 40%, 38%, and 46% of MPA alumni, respectively, completed the survey, and 36%, 38%, 34%, and 36% of the alumni of the 2-year MANP training programs, respectively, completed the survey. The number of practicing NPs and PAs has sharply increased since these professions were introduced in the Netherlands (Table 4) [5, 6, 27–33]. In 2012, 1442 NPs and 347 PAs were employed. Ten years later, 4,568 NPs and 1,590 PAs worked in the Netherlands. The intake in the training programs determines the size of both groups. There is no structural influx from abroad [1].

**Table 4:** Number of practicing NPs and PAs, full-time equivalent (FTE) per sector, and the ratio relative to every 100 FTE in specialized medical doctors, per January 1^a^

	2000	2006		2009	2012			2016			2019			2022		
	Practicing^b^				Prac	FTE	ratio^c^	Prac	FTE	ratio	Prac	FTE	ratio	Prac	FTE	ratio
**NPs**																
Hospital care						711	4.4		1,159	6.7		1,423	7.8		1,720	9
Primary care						75	0.9		149	1.7		197	2.1		260	2.5
(Nursing) home care						100	7.7		214	16		389	26.8		620	41.3
Mental healthcare^d^									671	23.6		938	32.2		1,347	41.9
Other^e^						233			140			120			160	
**Total**	**10**	**247**		**667**	**1,442**	**1,119**	**3.5**	**2,638**	**2,333**	**6.9**	**3,494**	**3,066**	**8.6**	**4,568**	**4,107**	**11**
**PAs**																
Hospital care						269	1.7		565	3.3		813	4.4		1,110	5.8
Primary care						26	0.3		58	0.7		91	1		160	1.6
(Nursing) home care^b^						11	0.8		32	2.4		27	1.9		80	5.3
Other						16			49			20			90	
**Total**		**15**		**149**	**347**	**323**	**1.0**	**762**	**704**	**2.1**	**1,058**	**951**	**2.7**	**1,590**	**1,450**	**3.9**

The alumni of the 3-year mental healthcare NP categorical training program have not been included in the 2012 study. In 2013, there were 398 NPs registered in mental healthcare.

^a^The reference dates of January 1 of the following year were used for the surveys we conducted at the end of 2018 and 2021.

^b^The numbers for the years 2000, 2006 and 2009 have a more significant uncertainty margin because they concern a retrospective request or a small group.

^c^Compared to FTEs in medical doctors in 2013, due to missing data for 2012.

^d^Includes all practicing NPs registered as mental healthcare NPs. Because the response categories in 2016 and 2019 were slightly different, the comparison is not entirely clear.

^e^In 2012, this included NPs working in home care, public health, and mental healthcare. These fields of work were not requested separately, or there were not enough respondents, to display data.

The capacity of NPs and PAs supersedes the growth of medical doctors in FTEs. While the number of FTEs in medical doctors increased from 2013 to 2022 by an annual growth rate of 2% [34], since 2012, the number of FTEs in NPs and PAs has increased annually by 14% and 16%, respectively. The capacity of NPs and PAs rose more steeply in all sectors than the capacity of medical doctors (Table 4). There were major differences between sectors in the capacity growth and the capacity ratio of NPs and PAs compared to medical doctors. For example, in 2012, 4.4 FTEs of NPs worked in hospital care relative to every 100 FTEs of medical doctors in this sector. In 2022 this ratio increased to 9.0/100. The capacity of NPs in hospital care increased by an annual growth rate of 9%. In (nursing) home care, the NP-medical doctor ratio increased from 7.7/100 FTE in 2012 to 41.3/100 FTE in 2022. For PAs, the number of FTEs grew fastest in (nursing) home care, at 22% per year, with the most significant increase (44% per year) after 2019. The PA-medical doctor ratio increased from 0.8/100 FTE in 2012 to 5.3/100 FTE in 2022. Approximately 85% of the NPs and 96% of the PAs in this sector work in nursing homes.

### Synthesis: Policy measures in relation to NP/PA workforce development

The moment of implementation of policy measures was compared with trends in the annual intake in training programs and the size of the professions. The results show that until 2012, the training program intake numbers were almost equal to the number of subsidized places. In 2012, a sharp increase in intake in the training programs coincided with the introduction of four policy measures: a temporary legal authorization for NP/PAs for specific medical procedures (extending the scope of practice), the inclusion of PAs in the Individual Health Care Professions Act (legal acknowledgment of the professional level), the start of an online knowledge platform, and a substantial increase in the number of subsidized places. The training intake in primary care increased in 2013 after an additional subsidy for training NPs, and PAs was awarded. The sharp decrease in the intake of NP/PA trainees in 2013 in hospitals, (nursing) home care, and mental healthcare coincided with covenants regarding fiscal austerity in these sectors.

From 2018–2019 we see a solid general increase in intake, especially in PA training programs in primary care. This finding coincides with the definitive inclusion of PAs in the Individual Health Care Professions Act, the definitive full authority of NP/PAs for specific medical procedures (extending the scope of practice), and primary care budget growth. From 2019, we also see a substantial increase in the number of FTEs of NP/PAs working in (nursing) home care and a shift in the NP-medical doctor and PA-medical doctor capacity ratios in (nursing) home care. This finding coincides with a new NP/PA reimbursement opportunity for extramural care to vulnerable groups in this sector. Other healthcare reimbursement policies do not coincide with NP/PA training or employment trends.

## Discussion

This paper provides an overview of the policy measures taken in the Netherlands to encourage task reallocation and to facilitate the training and deployment of NPs and PAs. We conducted a multimethod analysis of which policy measures coincide with trends in the workforce development of NPs and PAs. This paper reveals that some policy measures significantly coincide with and likely affected NP/PA workforce trends, while others do not consistently coincide with trends in the training and employment of NPs and PAs.

### The impact of training subsidies and fiscal austerity on the intake in training programs

Based on our research, we conclude that subsidies for NP and PA training strongly coincide with the intake in training programs and, thus, the development of the NP/PA workforce in the Netherlands. Nevertheless, the sharp decrease in the intake of NP/PA trainees in 2013, despite an increase in the number of subsidized training places, makes it clear that other factors in addition to the number of subsidized places may be important. For example, healthcare providers may have chosen to train fewer employees in 2013 due to ‘sudden’ fiscal austerity measures in 2012. A second alternative explanation for the decline is the increase in subsidized training places and intake in 2012. The number of subsidized places in previous years was possibly lower than the number of potential entrants in the programs, which led to an increasing reservoir of interested parties. After they started their training in 2012, the reservoir dried up. A third explanation, also suggested by Peters & Van der Horst (2016), is the lack of clarity within hospitals regarding the consequences of the planned introduction of a new reimbursement system in 2015 in hospitals, where hospitals now negotiate with insurance companies on the reimbursement of their self-employed physicians and collect these funds. This introduction led to an initial reluctance to train NPs and PAs since one of the uncertainties was who would employ these NPs and PAs [60].

However, although relevant, the second and third explanations do not clarify why the inflow declined the most in healthcare sectors, where fiscal austerity was also the most severe and not in primary care, which experienced higher budgetary growth. The influence of fiscal austerity on the number of newcomers in the programs remains the most likely explanation for the decrease in 2013. This position is further supported by the fact that the intake in hospital care, (nursing) home care, and mental healthcare sectors also decreased in most training programs, inducing a more general trend. A (temporary) decrease in the training of healthcare professionals has not yet been documented as a possible consequence of economic austerity. However, such an observation would certainly fit with the consideration of Visser et al. (2017) that high-yield interventions often require substantial investments, which proves to be an obstacle when providers are confronted with a decrease in financing [61]. Furthermore, the annual intake in NP and PA training programs within primary care increased sharply from the 2018–2019 cohort. This finding coincides with the agreements made in the 2018 covenant on continued budget growth in primary care [57]. Finally, over time the structural expansions in training subsidies seem to trump a one-off large fiscal contraction, although more of such contractions could lay ahead.

### High NP and PA employment in sectors with medical doctor shortages

Our results support earlier research in which medical doctor shortages are mentioned as one of the reasons for healthcare organizations to employ an NP or PA [15, 21, 22]. We showed considerable intersectoral differences in the ratio of FTEs in NPs versus FTEs in medical doctors. This ratio is relatively high in sectors with a high unfulfilled demand for medical doctors, such as care for disabled individuals, mental healthcare, and (nursing) home care [34]. Given the significant differences between sectors, it is plausible that exogenous factors, such as sectoral labor market shortages in medical doctors, accelerated the employment of NPs and PAs. From 2019, we see a substantial increase in NP/PA employment in nursing home care and entrants into PA training program in primary care. In 2019, an increasing shortage of elderly care physicians was signaled, as was a shortage of general practitioners in primary care for the first time since 2013. It is plausible that the increasing shortage of elderly care physicians accelerated the substantial increase in NP/PA employment in nursing home care. Another explanation is the introduction of new NP/PA reimbursement opportunities in 2019. However, other reimbursement regulations do not coincide with workforce or intake trends.

### Medical doctor shortages in relation to extending the scope of practice

In 2012, extending the scope of practice coincided with a sharp increase in the intake in training programs. Thus, extending the legal scope of practice might stimulate NP/PA workforce growth and professional roles, provided that certain situational conditions are met. For example, a collaborative relationship between a PA and a clinician is vital, as it builds trust followed by the number of reassigned tasks through negotiated performance autonomy [62, 63]. As De Bont et al. (2016) argued, role development is a situated endeavor in regard to extending the scope of practice. The development of such extended roles depends on, among other things, the willingness of local physicians to delegate tasks [64]. This willingness is probably closely related to regional and sectoral conditions, such as medical doctor capacity. Full practice authority is associated with a higher number of NPs in both rural areas and primary care, where there is a shortage of physicians [65, 66]. In combination with an extended scope of practice, medical doctor shortages may act as an accelerator for task reallocation and NP/PA workforce development. However, the results do not indicate an association because the 2012 intake increase also coincided with the inclusion of PAs in the Individual Health Care Professions Act, the start of an online knowledge platform, and an increase in the number of subsidized places. Because the 2012 intake increase in the training programs affects NPs and PAs, the inclusion of PAs in the Individual Health Care Professions Act is unlikely to be associated with the increase. It is also unlikely that the start of an online knowledge platform immediately impacts training program intake. In summary, the number of subsidized training places strongly coincides with the intake in training programs, leaving the role of the extended scope of practice unclear.

### Strengths and limitations

This is the first multimethod study to provide insight into the potential effects of various NP/PA policy measures on the workforce development of NPs and PAs in the Netherlands. In addition, the link between medical doctor capacity and the availability of rich data at the sector level provides further insight into the potential role of medical doctor capacity in NP/PA workforce development. A limitation of this study is that we must consider the possibility that the number of practicing (FTE in) NPs and PAs in the 2012 and 2016 studies has been overestimated. In these years, no bias was corrected for a somewhat higher nonresponse among nonpracticing graduates. The recent growth in the number of FTEs in PAs and NPs may be steeper than previously indicated. Another point of interest is that although this study shows that financial policy measures coincide with trends in the intake in training programs, there is no proof of causality. Although trends in education and employment do not coincide with other profession-specific policy measures, such as the funding of knowledge and consultation platforms and evaluation, these measures could still contribute to a gradual growth of the NP/PA workforce. A strong point of this research is that it adopts a multimethod approach on the national level in which, using multiple sources, a link has been made between government policy and NP/PA workforce development. Our study raises opportunities for future qualitative research to explain the potential causality of coinciding trends.

### Practical implications

Governments, health insurers, and other stakeholders need insight into the healthcare workforce and influencing factors. Such insight enables them to effectively create policies regarding the increasing demand for care, increasing healthcare costs, workforce shortages, and the deployment of new professions as possible solutions to such challenges. It is recommended that further qualitative research be conducted to better understand the differences between healthcare sectors in the scale of NP/PA employment and training and the potential causality of the coinciding trends described in this paper.

## Conclusions

This is the first longitudinal study on NPs and PAs to show that specific policies coincide with NP and PA workforce growth. We find that it is plausible that sudden and severe fiscal austerity has inhibited workforce growth. The internationally unique governmental subsidies for extensive master’s level training coincide and are likely associated with an increase in NP and PA training. Overall, we witnessed substantial NP/PA workforce growth over the years. In 2022, 4,568 NPs and 1,590 PAs were employed in the Netherlands, of whom the majority worked in hospital care, followed by mental healthcare (NPs), (nursing) home care, and primary care. The role of extending the scope of practice remains unclear. Other policy measures such as the legal acknowledgment of professional and educational levels, reimbursement, and funding knowledge platforms and research, do not consistently coincide with the trends in the intake in NP/PA training or employment. The differences in the scale of NP/PA employment between sectors can probably be largely explained by situational conditions.

## Supplementary Information

**Additional file 1: Appendix 1.** Background information on policy measures.

## Data Availability

The datasets used and/or analyzed during the current study are available from the corresponding author upon reasonable request.

## Abbreviations

ACMMP: Advisory Committee on Medical Manpower Planning (Stichting Capaciteitsorgaan in Dutch)
FTE: Full-time equivalent
MANP: Master Advanced Nursing Practice
MPA: Master Physician Assistant
NP: Nurse practitioner
PA: Physician assistant/physician associate
