# Supplementary material for: The influence of government policies on the nurse practitioner and physician assistant workforce in the Netherlands, 2000–2022: a multimethod approach study
Source: BMC Health Serv Res. 2023 Jun 6;23:580. doi: 10.1186/s12913-023-09568-4 (PMC10242803; doi:10.1186/s12913-023-09568-4)
Supplement: Supplementary file 1 — Additional file 1: Appendix 1. Background information on policy measures. [file 12913_2023_9568_MOESM1_ESM.docx]

# Appendix 1 Background information policy measures

Over the past decades, the Dutch government and health insurers have implemented various policy measures that may have affected NP/PA training and employment. The introduction of these policies followed the recommendations of the Committee Implementation Training Continuum and Task Reallocation in 2003 (1). Objectives for these policies are to contribute to the quality, accessibility, and affordability of care, as task reallocation contributes to a more efficient division of tasks between care professionals while offering career perspectives (2). Below we elaborate on these policies.

### Training subsidies

Both the 2-year MANP training and the 2.5-year MPA training were jointly funded by the Ministry of Education, Culture and Science and the Ministry of Health, Welfare, and Sport (3, 4). This subsidy scheme partly reimburses the salary costs to replace the trainee while s/he is following the course. There is also a reimbursement for the in-school training program. Since 2004, the number of subsidized training places has greatly increased (Table 2). After 2013 the number of structurally subsidized places was fixed at 250 places for the MPA and 450 for the MANP. The objectives of the subsidy scheme concern a more efficient organization, a better quality of care and a sufficient supply on the healthcare labor market, as task reallocation reduces the demand for physicians and offers career perspectives (5). In 2013, the “Strengthening NP and PA Training General Practice” project started, in which general practices and out-of-hours primary care services can apply for additional funding (6). Several studies have been carried out into the required (subsidized) training capacity in relation to the demand from the healthcare sector (7-12). In 2021, it was decided to finance training for 20 additional PAs within primary care (13). According to the most recent evaluation of the national subsidy scheme, the policy objectives are achieved. More NP/PA use justifies policies since: 1) PAs and NPs take over tasks from doctors, increasing the quality of care, 2) the introduction of these professions offers nurses and paramedics career prospects, 3) the subsidy scheme contributes to workforce growth because an increasing number of healthcare professionals can take over tasks from doctors (14).

### Funding platforms and research

Since 2004, the Dutch government has financed a knowledge center and consultation platforms on task reallocation. Additionally, research on task reallocation was funded (15-24). Several studies have been conducted on factors hindering or stimulating NP/PA deployment (25-30). The results of these investigations are partly the basis of several subsequent policies that the Dutch government took to remove obstacles to NP/PA deployment.

### Legal acknowledgment

Another policy measure concerned the inclusion of the professions in the Individual Health Care Professions Register. The purpose of the 1993 Individual Health Care Professions Act, which the register falls under, is to ensure that the healthcare quality is and remains high. The act also protects patients against incompetent and careless actions by healthcare providers (31). The government decision to include the PAs and NPs in the Individual Health Care Professions Act follows the advice of the Committee Implementation Training Continuum and Task Reallocation. The Committee recommended in 2003 that PAs and NPs should be included in the act so that: 1) an adequate training program could be set up and, 2) an independent legal authority could be arranged to make task reallocation possible. For the independent legal authority, it was recommended to start with a temporary arrangement (see 2012 in Table 2) (1).

The NP profession was included as a legally recognized specialism in 2009. The Dutch variant ‘nursing specialist’ [‘verpleegkundig specialist’] replaced the name Nurse Practitioner. The profession of PA followed in September 2018 and was included in the Individual Health Care Professions Act under Articles 3 and 33a and b. These professions obtained, among others, a protected professional title and governance by disciplinary law. Since January 1, 2014, and September 1, 2016, NP and PA graduates receive an MSc degree.

### Extending the scope of practice

From January 1, 2012, the scope of practice was extended for an initial period of five years. NPs and PAs have been granted legal authority for specific reserved medical procedures. Here, extending the scope of practice concerns routinely performed medical procedures of limited complexity, in which the risks are reasonable to oversee and for which national guidelines, standards, and protocols apply (32, 33). An evaluation showed that more NPs and PAs started to perform these reserved medical procedures. Moreover, they performed such procedures more autonomously and without consulting a physician. The amount of time they needed to carry out these procedures decreased significantly. The amended law was persistently considered an improvement by most NPs, PAs, and physicians (34, 35). From September 1, 2018, the extended autonomous scope of practice of NPs and PAs for specific reserved medical procedures became permanent. NPs and PAs can independently indicate, execute and delegate several reserved medical procedures depending on their experience and specialism and following laws and regulations, i.e., surgical procedures, endoscopies, catheterizations, injections, punctures, elective cardioversion, defibrillation, and prescribing drugs that are available only by prescription (34-37).

### Reimbursement regulations

The first reimbursement policy measure was taken in 2015 when the face-to-face criterion with a medical doctor for registration of an initial consultation was adjusted in hospital care. Since that year, NPs and PAs have been allowed to open and close tasks in the reimbursement system (38). As a result, they can carry out initial clinical visits independently in addition to follow-up visits. As of 2019, NPs and PAs may also register and invoice peer consultation and cotreatment activities in the reimbursement system under their name (39-41). Starting in 2022, hospital care activities must be registered in the reimbursement system under the profession code of the healthcare professional providing the care to promote task reallocation (42). In (nursing) home care and care for disabled individuals, starting in 2019, it became possible for NPs and PAs to provide and reimburse care for vulnerable groups in primary care, similar to elderly or disabled care physicians (43-45). In mental healthcare, PAs and NPs may provide and invoice care as coordinating practitioners (46-48). In addition, health insurers offer opportunities for providers to reimburse the deployment of NPs and PAs. In primary care, insurers make agreements about reimbursing projects or modules (49) (50).

### Healthcare funding

The Dutch government defines budgets for healthcare sectors where NPs and PAs operate. In the spring of 2012, agreements between the governing coalition and part of the opposition were made about an extensive program of fiscal austerity that would also affect curative care (among others, hospital care), long-term care (among others (nursing) home care), and mental healthcare (51). In the following years, the government made covenants with sectoral and professional organizations about, among others, healthcare budgets (Table 2) (52-58).

## References

1. LeGrand-van den Boogaard MJM, Erkelens DW, Kootstra G, Ros P, Schnabel P, Schulkes-van de Pol JA, et al. De zorg van morgen. Flexibiliteit & Samenhang. Deel 1 hoofdlijnen. Advies van de Commissie Implementatie Opleidingscontinuüm en Taakherschikking. [Tomorrow's care. Flexibility & Cohesion. Part 1 main features. Advice from the Commission Implementation Training Continuum and Task Reallocation.]. Den Haag; 2003. [23-7-2022]. Available from: <https://docplayer.nl/58286445-De-zorg-van-morgen-flexibiliteit-samenhang.html>.

2. Ministry of Health Welfare and Sport. Letter of the Minister of Health, Welfare, and Sport. The Hague 2003. Report No.: 29 282. [09-01-2020]. Available from: <https://www.parlementairemonitor.nl/9353000/1/j9vvij5epmj1ey0/vi3aloex17yx>

3. Subsidieregeling opleiding tot advanced nurse practitioner en opleiding tot physician assistant [Subsidy scheme for advanced nurse practitioner training and physician assistant training]. MEVA/BO-2720821 (2006). [10-07-2022]. Available from: <https://wetten.overheid.nl/BWBR0020517/2017-07-01>.

4. Jooren S, van Uden D, Leij-Halfwerk S, Jans L, van den Brink G. A Case Study Exploring Perceptions About Diversity in Higher Education Related to a Dutch Physician Assistant Program. The Journal of Physician Assistant Education. 2021;32(3):195-9. Available from: <https://journals.lww.com/jpae/Fulltext/2021/09000/A_Case_Study_Exploring_Perceptions_About_Diversity.15.aspx>.

5. Ecorys S. Taakherschikking: een toepassing via de opleiding van Verpleegkundig Specialisten en Physician Assistants. Eindrapportage beleidsdoorlichting Artikel 4.2 thema 3B. [Task reallocation: an application through the training of Nurse Practitioners and Physician Assistants. Final report policy review Article 4.2 theme 3B.]. 2016. [15-5-2021]. Available from: <https://www.google.com/url?sa=t&rct=j&q=&esrc=s&source=web&cd=&ved=2ahUKEwjQuoC968vwAhUh5OAKHTn5BLgQFjAAegQIAhAD&url=https%3A%2F%2Fwww.tweedekamer.nl%2Fdownloads%2Fdocument%3Fid%3D7fd973ae-1fd2-47d1-b8d8-2bfca54edab7%26title%3DTaakherschikking%253A%2520een%2520toepassing%2520via%2520de%2520opleiding%2520van%2520Verpleegkundig%2520Specialisten%2520en%2520Physician%2520Assistants.%2520Eindrapportage%2520beleidsdoorlichting%2520Artikel%25204.2%2520thema%25203B.pdf&usg=AOvVaw361kwEyEifw0A07uCahzJq>.

6. Stichting KOH. Stimuleringssubsidie 2022. [Incentive subsidy 2022.]. 2022 [10-07-2022]. Available from: <https://stichtingkoh.nl/taakherschikking/stimuleringssubsidie-2022>.

7. Capaciteitsorgaan. Capaciteitsplan 2021-2024. Deelrapport 9a. Physician Assistant [Capacity plan 2024-2027. Subreport 9a. Physician Assistant]. Utrecht: Stichting Capaciteitsorgaan; 2019. [07-01-2020]. Available from: <http://capaciteitsorgaan.nl/app/uploads/2019/12/Capaciteitsplan-2021-2024-Deelrapport-9a-Physician-Assistant.pdf>

8. Capaciteitsorgaan. Capaciteitsplan 2021-2024. Deelrapport 9b. Verpleegkundig Specialist Algemene Gezondheidszorg (AGZ) [Capacity plan 2024-2027. Subreport 9b. Nurse Practitioner General Healthcare.]. Utrecht: Stichting Capaciteitsorgaan; 2019. [ 07-01-2020]. Available from: <https://capaciteitsorgaan.nl/app/uploads/2019/12/Capaciteitsplan-2021-2024-Deelrapport-9b-Verpleegkundig-Specialist-AGZ.pdf>.

9. Kauffman K, Schuit H, Peters F. Resultaten Monitor NP-PA 2009-2010. Nijmegen: Kenniscentrum Beroepsonderwijs & Arbeidsmarkt; 2010. [10-07-2022]. Available from: <https://www.kbanijmegen.nl/doc/pdf/Monitor-NP-PA-2009-2010.pdf>.

10. Peters F, Keppels E, Schuit H, Kauffman K. De arbeidsmarkt- en opleidingsbehoefte aan nurse practitioners en physician assistants. [The labor market and training needs for nurse practitioners and physician assistants.]. Nijmegen: KBA Nijmegen; 2008. [10-07-2022]. Available from: <https://www.kbanijmegen.nl/doc/pdf/Arbeidsmarkt-opleidingsbehoefte-NP-PA.pdf>.

11. Peters F, Koenraadt G, Schuit H. De arbeidsmarkt van nurse practitioners en physician assistants. [The labor market of nurse practitioners and physician assistants.]. Nijmegen: KBA Nijmegen; 2007. [10-07-2022]. Available from: <https://www.kbanijmegen.nl/doc/pdf/nurse-practitioners_physician-assistants.pdf>.

12. Capaciteitsorgaan. Capaciteitsplan 2024-2027. Deelrapport 9. Physician Assistant en Verpleegkundig Specialist Algemene Gezondheidszorg. [Capacity plan 2024-2027. Subreport 9. Physician Assistant and Nurse Practitioner General Healthcare.]. Utrecht: Stichting Capaciteitsorgaan [Advisory Committee on Medical Manpower Planning]; 2022.

13. Riedstra S. Wind in de Zeilen. Voortgangsrapportage 15-05-2021 [Wind in the sails. Progress report 15-05-2021]. 2021. [03-06-2022]. Available from: <https://www.vlissingen.nl/inwoner/wind-in-de-zeilen/voortgangsrapportages/voortgangsrapportages-uitvoeringsregisseur.html>.

14. Peters F. Derde evaluatie van de ‘Subsidieregeling opleiding tot advanced nurse practitioner en opleiding tot physician assistant’ [Third evaluation of the 'Subsidy scheme for advanced nurse practitioner and physician assistant training']. Nijmegen: KBA Nijmegen; 2021. [15-09-2022]. Available from: <https://www.staten-generaal.nl/9370000/1/j4nvgs5kjg27kof_j9vvkfvj6b325az/vltvqk0wozzq>.

15. Raad voor de Volksgezondheid en Zorg. Taakherschikking in de gezondheidszorg. [Task reallocation in health care.]. Zoetermeer; 2002. [10-07-2022]. Available from: <https://www.raadrvs.nl/binaries/raadrvs/documenten/publicaties/2003/01/13/taakherschikking-in-de-gezondheidszorg/Advies_-_Taakherschikking_in_de_gezondheidszorg.pdf>.

16. Platform Zorgmasters. Over ons. [About us] [10-07-2022]. Available from: <https://zorgmasters.nl/over-ons/>.

17. Kenniscentrum Taakherschikking Eerste Lijn. Kenniscentrum Taakherschikking Eerste Lijn [Knowledge Center Task Reallocation Primary Care] [10-07-2022]. Available from: <https://stichtingkoh.nl/taakherschikking/kenniscentrum>.

18. Stuurgroep Taakherschikking Eerste Lijn. Voeden, bewaken en uitdragen. [Feeding, guarding and propagating.] [10-07-2022]. Available from: <https://stichtingkoh.nl/taakherschikking/kenniscentrum/stuurgroep>.

19. Dierick-van Daele A, Metsemakers J, Derckx E, Spreeuwenberg C, Vrijhoef HJM. Nurse practitioner in de huisartsenpraktijk. Onderzoeksrapport. [Nurse practitioner in general practice. Research report.]. Maastricht: Maastricht UMC+. 2008. [10-07-2022]. Available from: <https://a.storyblok.com/f/87251/x/4517ef5e99/rapport-nurse-practitioner-in-de-huisartsenpraktijk.pdf>.

20. De Leeuw J, Simkens A, Van Baar M, Van Balen F, Verheij R, Bijleveld S, et al. Taakherschikking in de huisartsenpraktijk: introductie van de Physician Assistant. Gevolgen voor de kwaliteit, de capaciteit en de kosten van de zorg. [Task reallocation in general practice: introduction of the Physician Assistant. Consequences for the quality, capacity and costs of care.]. Utrecht: UMC Utrecht; 2008. [10-07-2022]. Available from: <https://a.storyblok.com/f/87251/x/b8c49ccd3f/eindrapport-pa-huisartsenpraktijk-1.pdf>.

21. Van der Biezen M, Van der Burgt M, Laurant M. De physician assistant op de spoedpost. [The physician assistant at the emergency room.]. Radboudumc IQ Healthcare, Centrale Huisartsendienst Drenthe, Stichting KOH; 2017. [10-07-2022]. Available from: <https://a.storyblok.com/f/87251/x/2ff67ac349/rapport-de-physician-assistant-op-de-spoedpost.pdf>.

22. Van der Biezen M. The Impact of Nurse Practitioners in Primary Care [doctoral thesis]. Nijmegen 2017. [10-07-2022]. Available from: <https://www.iqhealthcare.nl/media/124514/the-impact-of-nurse-practitioners-in-primary-care-mieke-van-der-biezen.pdf>.

23. De Bruijn-Geraets DP. BIG issues: evaluation of the effects of granting legal authority to Dutch Nurse Practitioners and Physician Assistants to independently perform reserved medical procedures. [doctoral thesis]. Maastricht: Maastricht University; 2018. [10-07-2022]. Available from: <https://cris.maastrichtuniversity.nl/ws/portalfiles/portal/30558841/c6218.pdf>.

24. Lovink MH. The impact and organization of skill mix change in healthcare for older people. Substituting physicians with nurse practitioners, physician assistants or nurses. [doctoral thesis] 2019. [10-07-2022]. Available from: <https://www.ukonnetwerk.nl/media/1282/digitaal-proefschrift-marleen-lovink.pdf>.

25. Wallenburg I, Janssen M, Bont A. De rol van de verpleegkundig specialist en de physician assistant in de zorg. [The role of the nurse practitioner and the physician assistant in healthcare.]. 2015. [15-09-2022]. Available from: <https://www.eur.nl/sites/corporate/files/iBMG_2015_-_De_rol_van_de_Verpleegkundig_Specialist_en_de_Physician_Assistant_in_de_zorg_0.pdf>.

26. Kleven P, Leferink N, Van den Brink G, Kouwen A. De financiële effecten van taakherschikking. Een hermeting gericht op de effecten van de inzet van physician assistants en verpleegkundig specialisten in financieel-economisch perspectief. [The financial effects of task reallocation. A re-measurement aimed at the effects of the deployment of physician assistants and nurse practitioners from a financial-economic perspective.] Nijmegen: Hogeschool van Arnhem Radboudumc; 2019. [13-01-2020]. Available from: <https://zorgmasters.nl/extdocs/Eindrapport-De-financiele-effecten-van-taakherschikking-3-januari-2019.pdf>.

27. Lovink M, Van Vught A, Van den Brink G, Laurant M. Taakherschikking in de ouderenzorg: kansen, belemmeringen en effecten. [Task reallocation in elderly care: opportunities, obstacles and effects.]. Nijmegen: Radboudumc, IQ Healthcare & Eerstelijnsgeneeskunde. Hogeschool van Arnhem en Nijmegen, Lectoraat Organisatie van Zorg en Dienstverlening; 2017. [10-07-2022]. Available from: <https://www.iqhealthcare.nl/media/124512/20170908_eindrapportage-th-ouderenzorg_definitief.pdf>.

28. Van Klaveren S, Van der Meer E. De verpleegkundig specialist in de eerste lijn. Een verkenning van haar positie [Primary care nurse practitioner. A reconnaissance of her position]. Utrecht: BMC Advies. Hogeschool Utrecht; 2017 26-01-2017. [10-07-2022]. Available from: <https://www.bmc.nl/binaries/content/assets/bmcnl/pdfs/de-verpleegkundig-specialist-in-de-eerste-lijn.pdf>.

29. Timmermans M, Laurant M, Van Vught A. De effecten van het inzetten van Physician Assistants in de functie van zaalarts [The effects of using Physician Assistants in the function of ward physician]. Nijmegen: Radboudumc, IQ Healthcare; 2016. [10-07-2022]. Available from: <http://www.platformzorgmasters.nl/cms/wp-content/uploads/2016/12/Eindrapport-Worthy-Assistants-onderzoek.pdf>.

30. Kouwen A, Van den Brink G, Kleven P, Leferink N, Van Vught A, Grijzen Y. Taakherschikking en kostprijzen in de praktijk. Een meting gericht op de effecten van beleidsregelwijzigingen in 2015. [Task reallocation and cost prices in practice. A measurement focused on the effects of policy rule changes in 2015.]. 2016. [10-07-2022]. Available from: <https://zorgmasters.nl/extdocs/Taakherschikking-en-kostprijzen-in-de-praktijk-14-juli-2016.pdf>.

31. CIBG, Ministry of Health Welfare and Sport. Wet- en regelgeving. [Laws and regulations.]. n.d. [5-3-2023]. Available from: <https://www.bigregister.nl/registratie/nederlands-diploma-registreren/wet--en-regelgeving#:~:text=Het%20doel%20van%20de%20Wet,andere%20met%20het%20BIG%2Dregister>.

32. Tijdelijk besluit zelfstandige bevoegdheid verpleegkundig specialisten. [Temporary Decree on independent authorization for nurse practitioners.]. (2011). [10-07-2022]. Available from: <https://wetten.overheid.nl/BWBR0030980/2012-01-01>.

33. Tijdelijk besluit zelfstandige bevoegdheid physician assistant. [Temporary Decree on independent authorization for physician assistants.]. (2011). [10-07-2022]. Available from: <https://wetten.overheid.nl/BWBR0030978/2017-01-01>.

34. De Bruijn-Geraets DP, van Eijk-Hustings YJL, Bessems-Beks MCM, Essers BAB, Dirksen CD, Vrijhoef HJM. National mixed methods evaluation of the effects of removing legal barriers to full practice authority of Dutch nurse practitioners and physician assistants. BMJ Open. 2018;8(6):e019962. Available from: <https://www.ncbi.nlm.nih.gov/pubmed/29934382>.

35. De Bruijn-Geraets DP, Van Eijk-Hustings YJL, Castro-Van Soerland N, Vrijhoef HJM. voorBIGhouden 2. Eindrapportage Evaluatieonderzoek Art. 36a Wet BIG met betrekking tot de inzet van de Verpleegkundig Specialist en de Physician Assistant: Electieve cardioversie, defibrillatie, endoscopie. [voorBIGhouden 2. Final report Evaluation study Art. 36a BIG Act with regard to the deployment of the Nursing Practitioner and the Physician Assistant: Elective cardioversion, defibrillation, endoscopy.]. Maastricht UMC+, Patiënt & Zorg, KEMTA; 2016. [07-01-2020]. Available from: <http://venvnvs.nl/wp-content/uploads/sites/164/2016/12/2016-12-14-Rapport-voorBIGhouden-2.pdf>

36. 374. Wet van 4 oktober 2017, houdende wijziging van de Wet op de beroepen in de individuele gezondheidszorg in verband met het opnemen van de physician assistant in de lijst van registerberoepen, het toekennen van zelfstandige bevoegdheid voor bepaalde voorbehouden handelingen aan physician assistants en verpleegkundig specialisten en het opnemen van de mogelijkheid tot het instellen van een tijdelijk register voor experimenteerberoepen [374. Law of 4 October 2017, amending the Law on professions in individual health care in connection with the inclusion of the physician assistant in the list of registered professions, the granting of independent authority for certain reserved actions to physician assistants and nurse practitioners and the inclusion of the possibility to set up a temporary register for experimental professions.]. (2017). [10-07-2022]. Available from: <https://zoek.officielebekendmakingen.nl/stb-2017-374.pdf>.

37. Rijksoverheid. Welke voorbehouden handelingen mag een zorgverlener uitvoeren? [What reserved procedures may a healthcare provider perform?]. [18-08-2022]. Available from: <https://www.rijksoverheid.nl/onderwerpen/voorbehouden-handelingen/vraag-en-antwoord/voorbehouden-medische-handelingen>.

38. Regeling medisch specialistische zorg - NR/CU-260. [Regulations for specialist medical care - NR/CU-260.]. (2014). [10-07-2022]. Available from: <https://puc.overheid.nl/nza/doc/PUC_1839_22/>.

39. Regeling medisch-specialistische zorg - NR/REG-1907a. Versie 3. [Regulations on specialist medical care - NR/REG-1907a. Version 3.]. (2018). [10-07-2022]. Available from: <https://puc.overheid.nl/nza/doc/PUC_211874_22/>.

40. Nederlandse Zorgautoriteit. Monitor Taakherschikking. Physician assistants & Verpleegkundig specialisten in de medisch-specialistische zorg. [Monitor Task Reallocation. Physician assistants & Nurse practitioners in specialist medical care.]. 2019. [10-07-2022]. Available from: <https://puc.overheid.nl/nza/doc/PUC_268977_22/1/>.

41. Voorneveld-Nieuwenhuis J. NZa regelt ook DBC voor ICC en medebehandeling door VS en PA in het ziekenhuis. [Dutch healthcare authority also arranges DBC for ICC and co-treatment by NP and PA in the hospital.]. Dè Verpleegkundig Specialist. 2018. [10-07-2022]. Available from: <https://zorgmasters.nl/extdocs/DVS201804_10_NZa-regelt-ook-DBC-voor-ICC-en-medebehandeling-door-VS-en-PA-in-het-ziekenhuis_bvlgd.pdf>.

42. Taakherschikking. Update augustus 2021. Nuancering op artikel 23, lid 3 NR/REG-2207a; registratie AGB-code uitvoerend zorgverlener (RZ22a). [Task reallocation. Update August 2021. Qualification of Article 23, paragraph 3 NR/REG-2207a; registration AGB code executive care provider (RZ22a).]. (2021). [07-07-2022]. Available from: <https://www.nza.nl/zorgsectoren/medisch-specialistische-zorg/registreren-en-declareren-van-zorg/taakherschikking>.

43. Beleidsregel prestatiebeschrijvingen en tarieven modulaire zorg 2022 - BR/REG-22124c. [Policy rule for performance descriptions and rates for modular care 2022 - BR/REG-22124c.]. (2022). [07-07-2022]. Available from: <https://puc.overheid.nl/nza/doc/PUC_712174_22/1/>.

44. Beleidsregel Prestatiebeschrijvingen en tarieven modulaire zorg - BR/REG-19120b. [Policy rule Performance descriptions and rates for modular care - BR/REG-19120b.]. (2019). [21-7-2022]. Available from: <https://puc.overheid.nl/nza/doc/PUC_253088_22/2/>.

45. BIJLAGE 1 bij Beleidsregel prestatiebeschrijvingen en tarieven modulaire zorg 2022 - BR/REG-22124d. [Policy rule Performance descriptions and rates for modular care - BR/REG-19120b.]. [30-10-2022]. Available from: <https://eur02.safelinks.protection.outlook.com/?url=https%3A%2F%2Fpuc.overheid.nl%2FPUC%2FHandlers%2FDownloadBijlage.ashx%3Fpucid%3DPUC_723788_22_1%26bestand%3DBijlage_1_bij_BR-REG-22124d_Prestatiebeschrijvingen.pdf%26bestandsnaam%3DBijlage%2B1%2Bbij%2BBR-REG-22124d%2BPrestatiebeschrijvingen.pdf&data=05%7C01%7Cellen.dankers-demari%40radboudumc.nl%7C404dc779874146c9ae4908dab72e45d0%7Cb208fe69471e48c48d87025e9b9a157f%7C1%7C0%7C638023706774325249%7CUnknown%7CTWFpbGZsb3d8eyJWIjoiMC4wLjAwMDAiLCJQIjoiV2luMzIiLCJBTiI6Ik1haWwiLCJXVCI6Mn0%3D%7C3000%7C%7C%7C&sdata=TnKjKaWEbJU6qSsBfks7v32ZMB4pwiSjW64VlBKG7dg%3D&reserved=0>.

46. Beleidsregel Prestaties en tarieven geestelijke gezondheidszorg en forensische zorg - BR/REG-22137b [Policy rule Performance and rates for mental health care and forensic care]. [18-08-2022]. Available from: <https://puc.overheid.nl/nza/doc/PUC_658427_22/>.

47. Zorginstituut Nederland. Landelijk Kwaliteitsstatuut GGZ [National Mental Healthcare Quality Statute]. 2020. [07-07-2022]. Available from: <https://www.zorginzicht.nl/binaries/content/assets/zorginzicht/kwaliteitsinstrumenten/landelijk-kwaliteitsstatuut-ggz.pdf>.

48. Veldafspraak Tijdelijke invulling regiebehandelaarschap in 2022 [Field agreement Temporary implementation of coordinating practitioner in 2022]. 2021. [01-09-2022]. Available from: <https://www.zorgprestatiemodel.nl/shared/content/uploads/2021/12/Veldafspraak-Tijdelijke-invulling-regiebehandelaarschap-2022.pdf>.

49. Burgt M, Van Roij J, Derckx E, Meulepas M. Innovatie en Ondernemerschap. Hoe kan het dat de ene huisarts vier verpleegkundig specialisten in dienst heeft en de andere al moeite heeft om er één te financieren? [Innovation and Entrepreneurship. How is it possible that one general practitioner employs four nurse practitioners and the other is already struggling to finance one?]. Dé verpleegkundig specialist. 2016. [11].

50. LHV. Inzet personeel 2019. [Staff deployment 2019.]. [14-07-2022]. Available from: <https://meertijdvoordepatient.lhv.nl/voorbeelden/voorbeelden>.

51. ChristenUnie; CDA, D66; GroenLinks; VVD. Lenteakkoord: verantwoordelijkheid nemen in crisistijd. [Spring agreement: taking responsibility in times of crisis.]. 2012. [14-07-2022]. Available from: <https://docplayer.nl/2353034-Lenteakkoord-verantwoordelijkheid-nemen-in-crisistijd.html>.

52. Algemene Rekenkamer. Zorgakkoorden. Uitgavenbeheersing in de zorg deel 4. [Care agreements. Expenditure control in healthcare part 4.]. 2016. [14-07-2022]. Available from: <https://www.google.com/url?sa=t&rct=j&q=&esrc=s&source=web&cd=&ved=2ahUKEwiusqGklvj4AhWJzqQKHfVAD1kQFnoECBcQAQ&url=https%3A%2F%2Fwww.rekenkamer.nl%2Fbinaries%2Frekenkamer%2Fdocumenten%2Frapporten%2F2016%2F12%2F06%2Fzorgakkoorden%2FRapport%2BZorgakkoorden%2BWR.pdf&usg=AOvVaw3CHV-AOHwBsJZgJF2q_5nY>.

53. Ministerie van Volksgezondheid Welzijn en Sport, NVZ, NFU, Patiëntenfederatie Nederland, ZKN, FMS, et al. Addendum 2018 onderhandelaarsakkoord medisch-specialistische zorg 2014 t/m 2017. [Addendum 2018 negotiation agreement specialist medical care 2014 to 2017.]. 2017. [14-07-2022]. Available from: <https://www.eerstekamer.nl/overig/20170608/addendum_2018/document>.

54. Ministerie van Volksgezondheid Welzijn en Sport, LHV, InEen, Patiëntenfederatie Nederland, ZN. Addendum bestuurlijk akkoord huisartsenzorg en multidisciplinaire zorg 2018. [Addendum administrative agreement general practitioner care and multidisciplinary care 2018.]. 2017. Available from: <https://zoek.officielebekendmakingen.nl/blg-809178.pdf>.

55. De minister voor Medische Zorg en Sport, NVZ, NFU, Patiëntenfederatie Nederland, ZKN, FMS, et al. Bestuurlijk akkoord medisch-specialistische zorg 2019 t/m 2022. [Addendum administrative agreement general practitioner care and multidisciplinary care 2018.]. 2018. [14-07-2022]. Available from: <https://www.tweedekamer.nl/downloads/document?id=2c527fd9-1dce-4b18-9a8a-d09bc3e3986d&title=Bestuurlijk%20akkoord%20medisch-specialistische%20zorg%202019%20t%2Fm%202022.pdf>.

56. De minister voor Medische Zorg en Sport, LHV, InEen, Patiëntenfederatie Nederland, ZN. Bestuurlijk akkoord huisartsenzorg 2019 t/m 2022. [Administrative agreement for general practitioner care 2019 to 2022.]. 2018. [14-07-2022]. Available from: <https://www.tweedekamer.nl/downloads/document?id=0e57151d-cc64-4e6c-bd57-44e92002246c&title=Bestuurlijk%20akkoord%20huisartsenzorg%202019-2022.pdf>.

57. De staatssecretaris van Volksgezondheid Welzijn en Sport, NL GGZ, MIND, NVvP, LVVP, NIP, et al. Bestuurlijk akkoord geestelijke gezondheidszorg (GGZ) 2019 t/m 2022. [Administrative agreement on mental health care (GGZ) 2019 to 2022.]. 2018. [14-07-2022]. Available from: <https://www.tweedekamer.nl/downloads/document?id=443badd8-52f6-4abd-9d13-9b4fd3a78078&title=Bestuurlijk%20akkoord%20geestelijke%20gezondheidszorg%20%28GGZ%29%202019-2022.pdf>.

58. Minister van VWS, ActiZ, BTN, Patiëntenfederatie Nederland, VNG, V&VN, et al. Hoofdlijnenakkoord wijkverpleging 2019 t/m 2022. [Outline agreement for district nursing 2019 to 2022.]. 2018. [14-07-2022]. Available from: <https://www.tweedekamer.nl/downloads/document?id=642d504d-3197-4813-ad33-08c25fd8fa19&title=Hoofdlijnenakkoord%20wijkverpleging%202019-2022%20.pdf>.
